# Identification and longitudinal evaluation of inhaled medication adherence barriers using clinical natural language processing in asthma: a real-world cohort study

**DOI:** 10.3389/fmed.2026.1925068

**Published:** 2026-08-26

**Authors:** Chun-juan Zhang, Jia-hong Lu, Xiu-juan Ma, Gang Qiu, Min-feng Zhou

**Affiliations:** Haiyan People’s Hospital, Affiliated Haiyan Hospital of Jiaxing University, Jiaxing, Zhejiang, China

**Keywords:** adherence barriers, asthma, clinical natural language processing, longitudinal cohort, medication adherence, real-world study

## Abstract

**Background:**

To develop a rule-enhanced clinical natural language processing (clinical NLP) framework for the automated identification of inhaled medication adherence barriers in asthma, characterize their longitudinal trajectories, and evaluate their associations with longitudinal medication adherence.

**Methods:**

This real-world longitudinal cohort study included patients with asthma receiving standardized management at a tertiary hospital. Free-text telephone follow-up records from three visits were analyzed using a rule-enhanced clinical NLP framework to identify five adherence barriers: intentional treatment discontinuation, concerns about side effects, medication access barriers, unintentional forgetfulness, and disease denial. Generalized estimating equations (GEE) were used to assess longitudinal associations between adherence barriers and high medication adherence. Complete-case analysis and Firth penalized logistic regression were performed as sensitivity analyses.

**Results:**

A total of 216 patients were included; evaluable adherence data were available for 209, 194, and 176 patients at Waves 1, 2, and 3, respectively. The primary GEE analysis included 579 observations from 216 patients. The NLP framework achieved an overall accuracy of 97.4% and an F1 score of 0.883. Intentional treatment discontinuation (OR = 0.143, 95% CI: 0.080–0.258), concerns about side effects (OR = 0.330, 95% CI: 0.163–0.666), and unintentional forgetfulness (OR = 0.059, 95% CI: 0.012–0.278) were associated with lower odds of high adherence. Medication access barriers and disease denial were not statistically significant. Intentional treatment discontinuation increased across follow-up waves, whereas concerns about side effects increased mainly at the final wave. The proportion of low adherence increased from 11.5% among patients without identified barriers to 68.8% among those with three or more barriers (*p* for trend <0.001). Sensitivity analyses supported the associations for intentional treatment discontinuation and unintentional forgetfulness, whereas the association for concerns about side effects was less stable.

**Conclusion:**

Rule-enhanced clinical NLP may help extract adherence-related information from routine asthma follow-up records. Intentional treatment discontinuation and unintentional forgetfulness were relatively consistent markers of low adherence, whereas findings for concerns about side effects require cautious interpretation. External validation in larger, multi-center populations is needed before broader clinical application.

## 1 Introduction

Asthma is a heterogeneous chronic inflammatory airway disease that affects more than 260 million people worldwide. Inhaled corticosteroids (ICS), either alone or in combination with long-acting β2-agonists (LABAs), remain the cornerstone of long-term controller therapy. Nevertheless, suboptimal adherence to inhaled medication is common in routine clinical practice, with studies reporting that approximately 30–70% of patients do not use their medications as prescribed (1, 2). Inadequate adherence has been associated with poorer asthma control, more frequent exacerbations, greater healthcare utilization, and reduced quality of life (3). Identifying and understanding adherence-related difficulties is therefore an important component of long-term asthma management (4).

Medication adherence is commonly assessed using self-reported questionnaires (5), pharmacy refill measures such as the medication possession ratio or proportion of days covered, and electronic monitoring devices (6). Although these approaches provide useful information, each has limitations. Self-reported measures may be affected by recall and social desirability bias, pharmacy refill data indicate medication acquisition rather than actual use, and electronic monitoring devices may be costly or unavailable in routine practice (7). During routine follow-up, patients often describe their medication experiences, concerns, and difficulties in free-text conversations, providing potentially valuable information on adherence-related behaviors. However, these unstructured clinical narratives have rarely been systematically analyzed because of the complexity and variability of natural language.

Recent advances in clinical natural language processing (clinical NLP) have enabled the extraction of structured information from unstructured clinical text and have been increasingly applied to electronic health records and clinical documentation (8). Nevertheless, the application of clinical NLP to identify medication adherence barriers from longitudinal asthma follow-up records remains limited.

In this initial single center study, we developed and validated a rule-enhanced clinical NLP framework for identifying five predefined adherence barriers from Chinese-language routine follow-up records of patients with asthma. We further described the prevalence and longitudinal patterns of these barriers and examined their longitudinal associations with medication adherence. Because adherence status and barrier-related expressions were assessed within routine follow-up records, the identified barriers were considered correlates or behavioral markers of adherence rather than temporally established predictors. This study was intended to provide an initial assessment of the feasibility and interpretability of this approach, while recognizing that external validation is required before broader clinical application.

## 2 Methods

### 2.1 Study design and participants

This single center, real-world longitudinal cohort study included patients with physician-diagnosed asthma who received standardized management at the Department of Respiratory Medicine between November 2023 and June 2026. Asthma was diagnosed according to the Global Initiative for Asthma criteria. Routine assessments were scheduled at Wave 1, Wave 2 (3 months), and Wave 3 (6 months), during which medication adherence, Asthma Control Test (ACT) scores, and free-text follow-up records were collected.

Patients were eligible if they: (1) had a confirmed diagnosis of asthma; (2) received standardized inhaled therapy; (3) completed at least two evaluable follow-up visits; and (4) had corresponding free-text records available for NLP analysis. Patients with cough-variant asthma, chronic obstructive pulmonary disease, or other chronic respiratory diseases were excluded. Patients without at least two visits containing evaluable adherence data were excluded. Adherence status and barrier-related expressions were generally assessed during the same follow-up encounter. The barriers were therefore treated as contemporaneous correlates rather than temporally prior predictors. The follow-up completeness and sample included in the longitudinal analysis were presented in Supplementary Table S1.

### 2.2 Data collection and variable definitions

Demographic characteristics, ACT scores, medication information, adherence status, and free-text follow-up records were extracted from the electronic follow-up system. Medication adherence was assessed by trained respiratory physicians during telephone follow-up and classified as high adherence (regular medication use without missed doses or self-discontinuation) or low adherence (missed doses, self-reduction, self-discontinuation, or treatment interruption). ACT scores ranged from 5 to 25, with scores ≥20 indicating well-controlled asthma.

### 2.3 Identification of adherence barriers using clinical NLP

A rule-enhanced clinical natural language processing (clinical NLP) framework was developed to identify adherence barriers from free-text follow-up records. After text preprocessing, including text normalization and data cleaning, a keyword dictionary was constructed based on asthma guidelines, published literature, and expert consensus. Regular expression matching was then applied to identify predefined adherence barriers. Representative keywords, expressions, and classification rules are provided in Supplementary Table S2.

Five adherence barriers were defined: (1) intentional treatment discontinuation, (2) concerns about side effects, (3) medication access barriers, (4) unintentional forgetfulness, and (5) disease denial. Patients were considered positive for a barrier if the corresponding keywords were identified in any follow-up record. A Barrier Score (range: 0–5) was calculated as the total number of identified barriers for graded association analysis. To validate the NLP framework, 50 randomly selected patients were independently annotated by two respiratory physicians, with two records sampled per patient. Disagreements were resolved through discussion, and consensus annotations were used as the reference standard. Inter-physician agreement was assessed using percentage agreement and Cohen’s *κ*. NLP performance was evaluated using accuracy, positive predictive value, sensitivity, specificity, and F1 score, with overall performance micro-pooled across the five barrier categories. The validation results are provided in Supplementary Table S3. The overall inter-physician Cohen’s *κ* was 0.914. Compared with the physician consensus reference standard, the NLP framework achieved an overall accuracy of 97.4%, sensitivity of 96.1%, specificity of 97.6%, positive predictive value of 81.7%, and F1 score of 0.883.

### 2.4 Longitudinal data construction

Data from the three follow-up visits were converted into long format, with each patient contributing multiple observations. The longitudinal dataset included patient identifier, follow-up visit, age, sex, adherence status, ACT score, and the five adherence barriers. Patient identifier was specified as the clustering variable to account for within-subject correlations.

### 2.5 Statistical analysis

Continuous variables are presented as mean ± standard deviation (SD), and categorical variables as frequencies and percentages. Between-group comparisons were performed using the Mann–Whitney U test for continuous variables and the *χ*^2^ test or Fisher’s exact test for categorical variables. Longitudinal associations between adherence barriers and high adherence were evaluated using generalized estimating equations (GEE) with a logit link and an exchangeable working correlation structure. The primary model simultaneously included all five adherence barriers and was adjusted for follow-up wave and age. Results are reported as odds ratios (ORs) with 95% confidence intervals (95% CIs). Two sensitivity analyses were performed: (1) Firth penalized logistic regression to evaluate the association between adherence barriers and adherence status at the final follow-up, and (2) complete-case analysis by repeating the GEE model among patients with complete follow-up data. All analyses were performed using R version 4.3.3 (R Foundation for Statistical Computing, Vienna, Austria). A two-sided *p* value <0.05 was considered statistically significant.

## 3 Results

### 3.1 Baseline characteristics

At Wave 1, adherence data were available for 209 patients, including 202 with high adherence and 7 with low adherence. The mean age was 49.1 ± 16.1 years, 52.2% were female, and the mean ACT score was 23.6 ± 0.9. Age, sex, and ACT score did not differ between adherence groups. Disease denial was more frequent in the low-adherence group (71.4% vs. 21.3%; *p* = 0.008), whereas the other barrier categories showed no significant differences (Table 1). At Wave 3, 176 patients had evaluable adherence data, including 114 with high adherence and 62 with low adherence. The high-adherence group had a slightly higher ACT score (24.7 ± 0.6 vs. 24.3 ± 1.0; *p* = 0.031), while intentional treatment discontinuation, concerns about side effects, unintentional forgetfulness, and disease denial were more frequent in the low-adherence group; medication access barriers did not differ significantly (Supplementary Table S4). Wave 1 comparisons should be interpreted cautiously because only seven patients had low adherence.

**Table 1:** Patient characteristics and cumulative adherence barriers according to adherence status at Wave 1.

Variable	Total (*n* = 209)	High (*n* = 202)	Low (*n* = 7)	*p* value
Age (years)	49.1 ± 16.1	48.9 ± 16.0	53.7 ± 18.6	0.546
ACT Score	23.6 ± 0.9	23.6 ± 0.9	24.0 ± 1.0	0.373
Sex: Female	109 (52.2%)	105 (52.0%)	4 (57.1%)	1.000
Male	100 (47.8%)	97 (48.0%)	3 (42.9%)	
Barrier: Intentional stop	71 (34.0%)	68 (33.7%)	3 (42.9%)	0.921
Barrier: Side-effect fear	35 (16.7%)	34 (16.8%)	1 (14.3%)	1.000
Barrier: Access barrier	76 (36.4%)	73 (36.1%)	3 (42.9%)	1.000
Barrier: Unintentional forget	14 (6.7%)	13 (6.4%)	1 (14.3%)	0.962
Barrier: Disease denial	48 (23.0%)	43 (21.3%)	5 (71.4%)	0.008

Table included 209 patients with evaluable adherence data at Wave 1. Barrier variables were patient-level cumulative indicators. A patient was classified as positive if the corresponding barrier was identified in at least one available follow-up text. These cumulative variables were used for descriptive purposes and were distinct from the visit-specific barrier indicators used in the GEE analysis.

### 3.2 Overall prevalence of adherence barriers during follow-up

**Figure 1:**
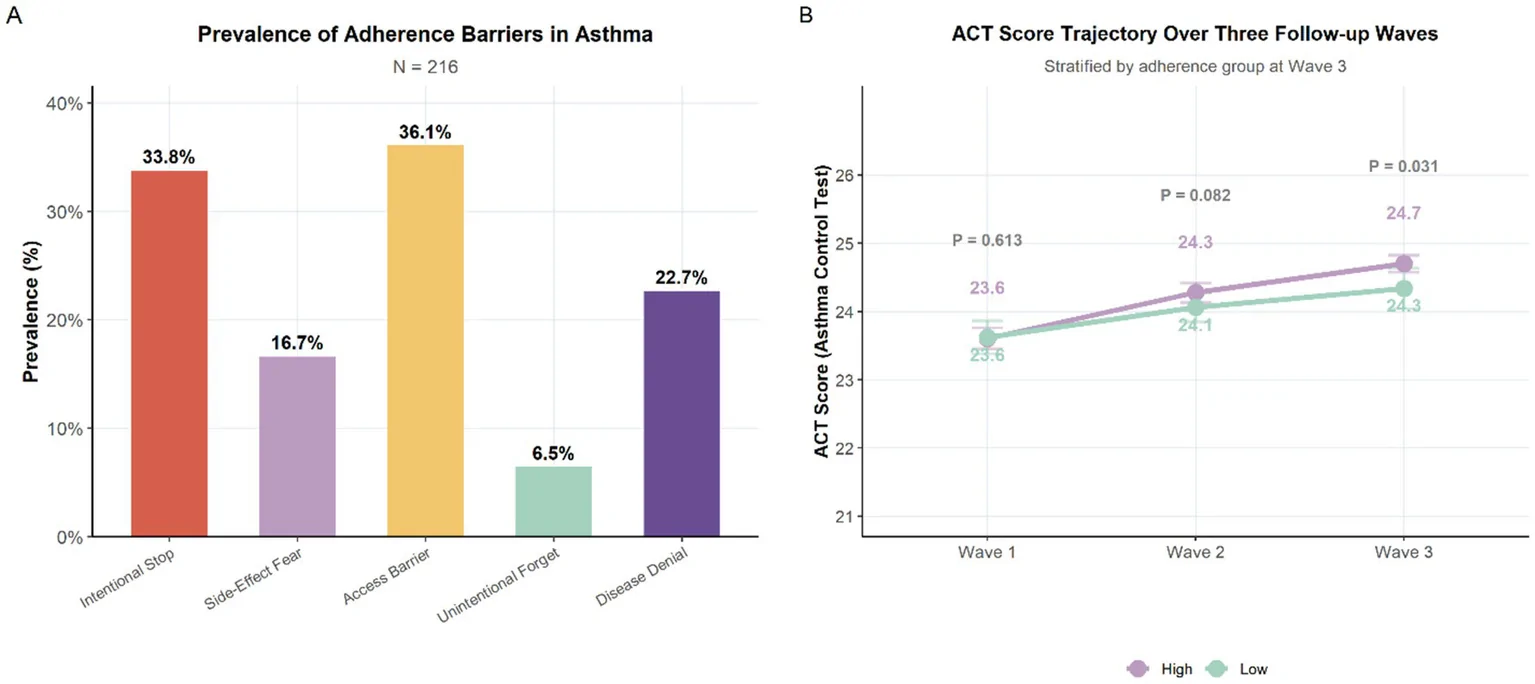
Patient-level cumulative prevalence of five medication adherence barriers **(A)** and ACT score trajectories stratified by Wave 3 adherence status **(B)**.

Among the 216 patients included in the revised longitudinal cohort, medication access barriers were the most prevalent adherence barrier during follow-up (36.1%), followed by intentional treatment discontinuation (33.8%), disease denial (22.7%), concerns about side effects (16.7%), and unintentional forgetfulness (6.5%) (Figure 1A). As shown in Figure 1B, ACT scores were comparable between the high- and low-adherence groups. When patients were stratified by Wave 3 adherence status, ACT scores were similar between groups at Waves 1 and 2 (both *p* > 0.05), whereas the high-adherence group had a slightly higher ACT score at Wave 3 (*p* < 0.05).

### 3.3 Longitudinal patterns of adherence barriers and graded association with low adherence

**Figure 2:**
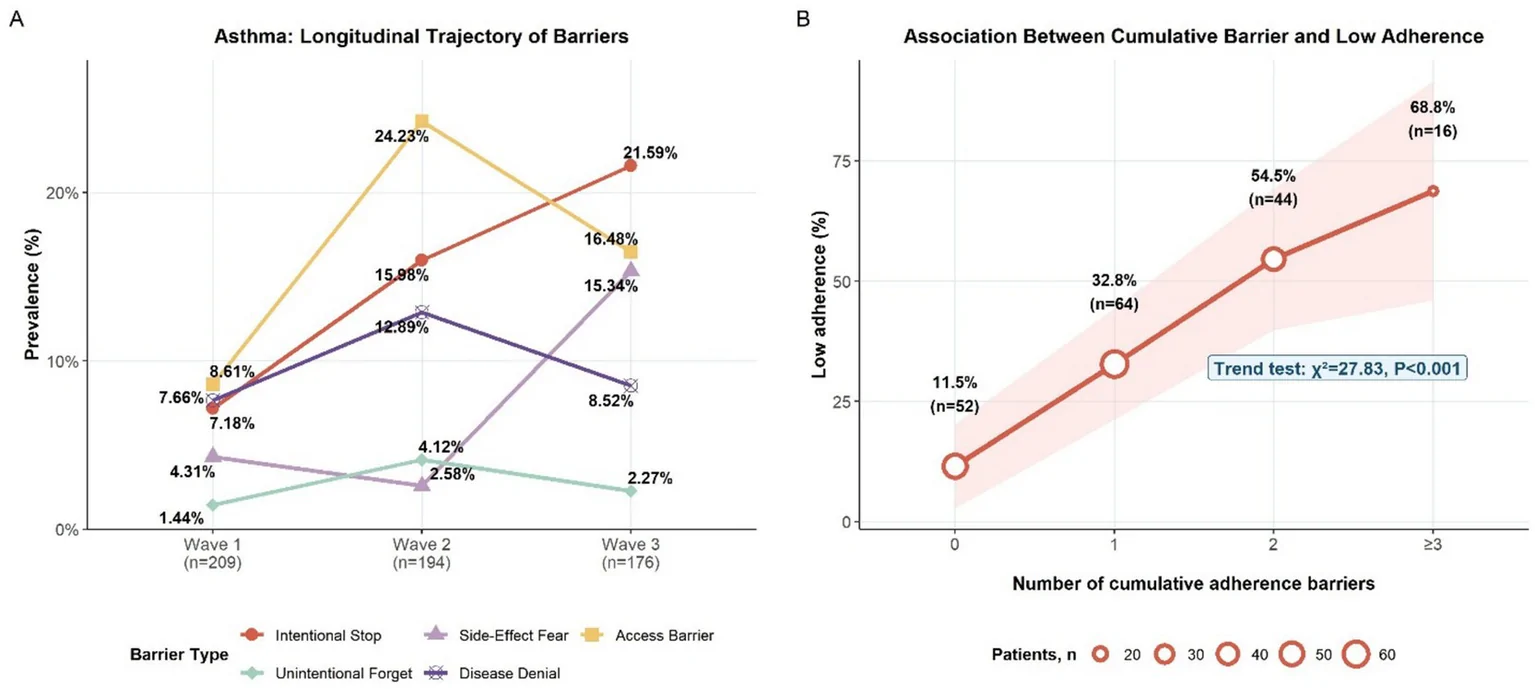
Visit-specific prevalence of adherence barriers and their graded association with low adherence. **(A)** Prevalence of five adherence barriers across the three follow-up waves. **(B)** Association between cumulative barrier count and low adherence at Wave 3.

The prevalence of adherence barriers varied across the three follow-up waves (Figure 2A). Intentional treatment discontinuation increased progressively from 7.2% at Wave 1 to 16.0% at Wave 2 and 21.6% at Wave 3. Medication access barriers increased from 8.6% at Wave 1 to a peak of 24.2% at Wave 2, before declining to 16.5% at Wave 3, while remaining higher than at Wave 1. Concerns about side effects were relatively uncommon at Waves 1 and 2 (4.3 and 2.6%, respectively) but increased to 15.3% at Wave 3. Disease denial increased from 7.7% at Wave 1 to 12.9% at Wave 2 and subsequently declined to 8.5% at Wave 3. Unintentional forgetfulness remained infrequent throughout follow-up, with rates of 1.4, 4.1, and 2.3% across the three waves, respectively. A significant graded association was observed between the cumulative number of adherence barriers and low adherence at Wave 3 (Figure 2B). The proportion of patients with low adherence increased from 11.5% (6/52) among those without identified barriers to 32.8% (21/64), 54.5% (24/44), and 68.8% (11/16) among those with one, two, and three or more barriers, respectively (P for trend <0.001).

### 3.4 Longitudinal association between adherence barriers and medication adherence

In the GEE model including all five predefined adherence barriers, intentional treatment discontinuation (OR = 0.143, 95% CI: 0.080–0.258; *p* < 0.001), concerns about side effects (OR = 0.330, 95% CI: 0.163–0.666; *p* = 0.002), and unintentional forgetfulness (OR = 0.059, 95% CI: 0.012–0.278; *p* < 0.001) were associated with lower odds of high adherence. Medication access barriers (OR = 0.955, 95% CI: 0.503–1.812; *p* = 0.887) and disease denial (OR = 0.559, 95% CI: 0.238–1.311; *p* = 0.181) were not independently associated with adherence. High adherence also decreased across follow-up waves (OR = 0.279, 95% CI: 0.207–0.377; *p* < 0.001), whereas the association with age did not reach statistical significance (OR = 0.983, 95% CI: 0.966–1.001; *p* = 0.060) (Table 2).

**Table 2:** Longitudinal associations between NLP-derived adherence barriers and high medication adherence.

Variable	OR	95% CI	*p* value
Intentional stop	0.143	0.080–0.258	<0.001
Side effects fear	0.330	0.163–0.666	0.002
Access barriers	0.955	0.503–1.812	0.887
Unintentional forgetfulness	0.059	0.012–0.278	<0.001
Disease denial	0.559	0.238–1.311	0.181
Follow-up Wave	0.279	0.207–0.377	<0.001
Age, years	0.983	0.966–1.001	0.060

### 3.5 Sensitivity analyses

Sensitivity analyses provided further support for the associations of intentional treatment discontinuation and unintentional forgetfulness with lower odds of high medication adherence (Table 3). In the Firth penalized logistic regression based on the final follow-up data, intentional treatment discontinuation (OR = 0.253, 95% CI: 0.117–0.530; *p* < 0.001), concerns about side effects (OR = 0.164, 95% CI: 0.064–0.399; *p* < 0.001), and unintentional forgetfulness (OR = 0.066, 95% CI: 0.010–0.303; *p* < 0.001) were associated with lower odds of high adherence. Disease denial and medication access barriers were not statistically significant.

**Table 3:** Sensitivity analyses of the associations between adherence barriers and high medication adherence.

Variable	Firth logistic regression		Complete-case GEE	
	OR (95% CI)	*p* value	OR (95% CI)	*p* value
Adherence barriers				
Intentional stop	0.253 (0.117–0.530)	<0.001	0.177 (0.089–0.356)	<0.001
Side-effect fear	0.164 (0.064–0.399)	<0.001	0.438 (0.174–1.101)	0.079
Unintentional forgetfulness	0.066 (0.010–0.303)	<0.001	0.079 (0.013–0.471)	0.005
Disease denial	0.463 (0.193–1.109)	0.084	0.415 (0.144–1.195)	0.103
Access barrier	1.645 (0.752–3.737)	0.215	1.722 (0.806–3.678)	0.160
Covariates				
Follow-up wave	–	–	0.217 (0.145–0.323)	<0.001
Age (years)	0.982 (0.959–1.005)	0.121	0.986 (0.963–1.011)	0.270

OR, odds ratio; CI, confidence interval; GEE, generalized estimating equation. Firth logistic regression was conducted on the final follow-up data (wave 3) to mitigate small-sample bias. Complete-case GEE analysis was performed on longitudinal data restricted to patients with complete observations across all three follow-up waves.

In the complete-case GEE analysis restricted to patients with complete data across all three follow-up waves, intentional treatment discontinuation (OR = 0.177, 95% CI: 0.089–0.356; *p* < 0.001) and unintentional forgetfulness (OR = 0.079, 95% CI: 0.013–0.471; *p* = 0.005) remained associated with lower odds of high adherence. The association for concerns about side effects was attenuated and no longer reached statistical significance (OR = 0.438, 95% CI: 0.174–1.101; *p* = 0.079). Disease denial and medication access barriers remained nonsignificant. High adherence also decreased across follow-up waves (OR = 0.217, 95% CI: 0.145–0.323; *p* < 0.001), whereas age and sex were not significantly associated with adherence.

## 4 Discussion

In this real-world longitudinal cohort, we developed and internally validated a rule-enhanced clinical NLP framework to identify five predefined adherence barriers from routine asthma follow-up records. In the primary GEE model, intentional treatment discontinuation, concerns about side effects, and unintentional forgetfulness were associated with lower odds of high adherence, whereas medication access barriers and disease denial were not. The associations for intentional treatment discontinuation and unintentional forgetfulness were relatively consistent across sensitivity analyses. By contrast, the association for concerns about side effects was attenuated and no longer statistically significant in the complete-case analysis. The barriers also showed distinct temporal patterns, and a greater cumulative number of barriers was associated with a higher proportion of low adherence. The NLP framework showed favorable overall performance in the internal validation sample; however, category-specific estimates for concerns about side effects and unintentional forgetfulness were based on few positive records and should be interpreted cautiously.

Previous studies have mainly assessed asthma medication adherence using self-reported questionnaires, pharmacy refill records, or electronic monitoring devices (9, 10). Although electronic monitoring is often considered a reference method, its cost and operational requirements may limit routine use in long-term follow-up (11). Pharmacy refill records reflect medication acquisition rather than actual use, whereas self-reported measures are susceptible to recall and social desirability bias. In contrast, this study used routinely collected free-text follow-up records and required no additional data collection. By extracting adherence-related information from unstructured clinical narratives, clinical NLP may complement conventional adherence measures. The longitudinal design also allowed changes in adherence barriers to be described over time rather than at a single assessment.

Our findings are broadly consistent with previous studies reporting an association between intentional treatment discontinuation and lower medication adherence (12, 13). Patients may reduce or stop inhaled therapy after symptom improvement because of limited awareness of persistent airway inflammation or concerns about long-term treatment. This is consistent with the Global Initiative for Asthma recommendations, which emphasize continued controller therapy to maintain asthma control and reduce exacerbation risk. Concerns about side effects were also associated with lower adherence in the primary analysis and became more frequent at later follow-up, in keeping with previous reports on corticosteroid-related concerns (14, 15). However, this association was less stable in the complete-case analysis and should be interpreted cautiously. Unintentional forgetfulness was uncommon but remained associated with lower adherence across analyses (16, 17). These findings indicate that repeated assessment of medication-taking concerns and behaviors may be useful during long-term asthma follow-up.

Adherence barriers also varied over time. Intentional treatment discontinuation increased progressively, whereas concerns about side effects became more frequent at later visits. These patterns suggest that adherence-related difficulties may change during disease management and that repeated assessment may provide information not captured by a single cross-sectional evaluation (18, 19). We also observed a graded association between the number of adherence barriers and low adherence, suggesting that several behavioral or practical difficulties may coexist. However, the observational design does not establish causality or demonstrate that addressing multiple barriers would necessarily improve adherence. High adherence also declined across follow-up waves, consistent with previous longitudinal studies of long-term asthma management (20, 21). Together, these findings support continued assessment of adherence-related difficulties during routine follow-up.

Several limitations should be noted. First, this was a single center study with a modest sample size, which may limit generalizability. Although only patients with at least two evaluable visits were included, follow-up remained incomplete for some participants. Patients with poorer adherence may have been less likely to complete follow-up, and informative loss to follow-up cannot be excluded. Second, adherence status and barrier-related expressions were generally assessed during the same visit. Intentional discontinuation and forgetfulness also overlap partly with the operational definition of low adherence. Temporal precedence could therefore not be established, and reverse causation, conceptual overlap, and same-source measurement may have contributed to the observed associations. The identified barriers should thus be interpreted as contemporaneous behavioral markers rather than established predictors or causes. Third, the framework was developed from Chinese-language records at a single center and may depend on local terminology, documentation style, and clinical practice. It may not fully capture implicit meanings, complex semantics, or contextual negation. Local adaptation and external validation would therefore be required before use in other settings. Fourth, several barrier categories contained few positive records in the validation sample, resulting in imprecise category-specific estimates. Finally, education, socioeconomic status, inhaler technique, treatment complexity, and psychological factors were unavailable, and residual confounding remains possible.

## 5 Conclusion

In this single center longitudinal study, a rule-enhanced clinical NLP framework was used to identify adherence-related barriers from routine asthma follow-up records. Intentional treatment discontinuation and unintentional forgetfulness showed relatively consistent inverse associations with high adherence, whereas the association for concerns about side effects was less stable across analyses. A greater cumulative number of barriers was also associated with a higher proportion of low adherence. These findings provide an initial assessment of the potential value of clinical NLP for extracting adherence-related information from routine documentation. External validation in larger, multicenter, and more diverse populations is needed before broader clinical application.

## Data Availability

The raw data supporting the conclusions of this article will be made available by the authors, without undue reservation.

## References

[1] Hosseininia S Mohammadikebar S Motashakkeri L Kamran A. Determinants of adherence to inhaler use in patients with asthma: the role of knowledge, self-efficacy, and perceived barriers. BMC Pulm Med. (2025) 25:456. doi: 10.1186/s12890-025-03919-z, 41063043 PMC12505728

[2] Alqarni AA Aldhahir AM Siraj RA Alqahtani JS Alghamdi DA Alghamdi SK . Asthma medication adherence, control, and psychological symptoms: a cross-sectional study. BMC Pulm Med. (2024) 24:189. doi: 10.1186/s12890-024-02995-x, 38641584 PMC11031990

[3] Nie B Chapman SCE Chen Z Wang X Wei L. Utilization of the beliefs about medicine questionnaire and prediction of medication adherence in China: a systematic review and meta-analysis. J Psychosom Res. (2019) 122:54–68. doi: 10.1016/j.jpsychores.2019.03.184, 31006535

[4] Rackow P Drennan A Pinnock H Dima AL. Optimizing adherence to medication to improve outcomes in asthma. Curr Opin Pulm Med. (2025) 31:262–9. doi: 10.1097/MCP.0000000000001166, 40105049 PMC11957441

[5] Francis A Abraham E Verma G Mathew L Acharya S Kumar S . Assessment of knowledge and attitude of asthmatic patients and their caregivers regarding the disease using an asthma knowledge questionnaire in a tertiary care hospital in Chennai. Cureus. (2024) 16:e69832. doi: 10.7759/cureus.69832, 39435231 PMC11491873

[6] Zewdie S Bayked EM Ayenew W Seyfu A Andargie A. Prevalence and predictors of medication adherence among adolescents and adults with asthma in Ethiopia: a systematic review and meta-analysis. J Asthma. (2025) 62:905–18. doi: 10.1080/02770903.2024.2332920, 38512046

[7] Sasaki J Kawayama T Yoshida M Takahashi K Fujii K Machida K . Adherence barriers to inhaled medicines in Japanese older patients with asthma evaluated using the "Adherence Starts with Knowledge 20" (ASK-20) questionnaire. J Asthma. (2019) 56:632–41. doi: 10.1080/02770903.2018.1484132, 29969920

[8] Murff HJ FitzHenry F Matheny ME Gentry N Kotter KL Crimin K . Automated identification of postoperative complications within an electronic medical record using natural language processing. JAMA. (2011) 306:848–55. doi: 10.1001/jama.2011.1204, 21862746

[9] Stewart SF Moon Z Horne R. Medication nonadherence: health impact, prevalence, correlates and interventions. Psychol Health. (2023) 38:726–65. doi: 10.1080/08870446.2022.2144923, 36448201

[10] Zhang X Ding R Zhang Z Chen M Yin Y Quint JK. Medication adherence in people with asthma: a qualitative systematic review of patient and health professional perspectives. J Asthma Allergy. (2023) 16:515–27. doi: 10.2147/JAA.S407552, 37193110 PMC10182790

[11] Harada N Harada S Ito J Atsuta R Hori S Takahashi K. Mobile health app for Japanese adult patients with asthma: clinical observational study. J Med Internet Res. (2020) 22:e19006. doi: 10.2196/19006, 32795993 PMC7455863

[12] Hedenrud T Jakobsson A El Malla H Håkonsen H. "I did not know it was so important to take it the whole time" – self-reported barriers to medical treatment among individuals with asthma. BMC Pulm Med. (2019) 19:175. doi: 10.1186/s12890-019-0934-3, 31533679 PMC6751752

[13] Laforest L El Hasnaoui A Pribil C Ritleng C Osman LM Schwalm MS . Asthma patients’ self-reported behaviours toward inhaled corticosteroids. Respir Med. (2009) 103:1366–75. doi: 10.1016/j.rmed.2009.03.010, 19398316

[14] Cooper V Metcalf L Versnel J Upton J Walker S Horne R. Patient-reported side effects, concerns and adherence to corticosteroid treatment for asthma, and comparison with physician estimates of side-effect prevalence: a UK-wide, cross-sectional study. NPJ Prim Care Respir Med. (2015) 25:15026. doi: 10.1038/npjpcrm.2015.26, 26158805 PMC4497315

[15] Aggarwal B Al-Moamary M Allehebi R Alzaabi A Al‑Ahmad M Amin M . APPaRENT 3: asthma patients’ and physicians’ perspectives on the burden and management of asthma in seven countries. Adv Ther. (2024) 41:3089–118. doi: 10.1007/s12325-024-02900-2, 38874879 PMC11263244

[16] Al-Moamary M Aggarwal B Al-Ahmad M Sriprasart T Koenig S Levy G . Are treatment adherence factors apparent in patients with asthma and to physicians? Results from the APPaRENT 3 survey. Adv Ther. (2025) 42:1506–21. doi: 10.1007/s12325-025-03105-x, 39912987 PMC11868149

[17] Chebli AI Chelighem Z Zebbiche Y Abdennour S Amziane A Djidjik R. Factors associated with therapeutic non-adherence in asthmatic patients: a multicenter study in Algeria. Ann Pharm Fr. (2025) 83:367–77. doi: 10.1016/j.pharma.2024.10.010, 39486789

[18] Zewdie S Mekuria B Alemu BK Bayked EM NurAhmed Toleha H Ayenew W . Prevalence of medication adherence among adult asthmatic patients in four African countries: a systematic review and meta-analysis. World Allergy Organ J. (2024) 17:100870. doi: 10.1016/j.waojou.2024.100870, 38304621 PMC10831257

[19] Alenezi NM Alnajjar JS Alnajem AH Almuaybid RA Abuhomoud Z Al Ibrahim MH . Factors affecting medication and refill adherence among patients with asthma in Saudi Arabia: a stratified random cross-sectional study. BMC Pulm Med. (2025) 25:561. doi: 10.1186/s12890-025-04032-x, 41420231 PMC12717724

[20] Burkhart PV Sabaté E. Adherence to long-term therapies: evidence for action. J Nurs Scholarsh. (2003) 35:207–7. doi: 10.1111/j.1547-5069.2003.tb00001.x 14562485

[21] Chipps BE Haselkorn T Paknis B Ortiz B Bleecker ER Kianifard F . More than a decade follow-up in patients with severe or difficult-to-treat asthma: the epidemiology and natural history of asthma: outcomes and treatment regimens (TENOR) II. J Allergy Clin Immunol. (2018) 141:1590–1597.e9. doi: 10.1016/j.jaci.2017.07.014 28797732

