# Supplementary material for: Identification and longitudinal evaluation of inhaled medication adherence barriers using clinical natural language processing in asthma: a real-world cohort study
Source: Front Med (Lausanne). 2026 Aug 26;13:1925068. doi: 10.3389/fmed.2026.1925068 (PMC13553807; doi:10.3389/fmed.2026.1925068)
Supplement: Supplementary file 1 [file Supplementary_file_1.docx]

**Supplementary Table**

**Table S1. Follow-up completeness and sample included in the longitudinal analysis**

| **Follow-up characteristic** | **Patients, n (%)** |
| --- | --- |
| Revised longitudinal cohort | 216 (100.0) |
| Evaluable adherence data at Wave 1 | 209 (96.8) |
| Evaluable adherence data at Wave 2 | 194 (89.8) |
| Evaluable adherence data at Wave 3 | 176 (81.5) |
| Completed exactly two follow-up visits | 69 (31.9) |
| Completed all three follow-up visits | 147 (68.1) |
| Included in the primary GEE analysis | 216 patients |
| Visit-level observations included in the primary GEE analysis | 579 observations |

**Table S2. Representative keywords, expressions, and classification rules for five medication adherence barriers**

| **Barrier category** | **Representative Chinese expressions** | **English interpretation** | **Main classification rule** |
| --- | --- | --- | --- |
| Intentional treatment discontinuation | 自行停药；擅自减量；症状好转后停用；不想用 | Stopped or reduced medication intentionally | Positive when intentional stopping or reduction was explicitly documented |
| Concerns about side effects | 副作用；担心依赖；声音嘶哑 | Concern about adverse effects or dependence | Positive when medication avoidance was linked to actual or anticipated adverse effects |
| Medication access barrier | 太远；药物太贵；买不到；取药不便 | Difficulty obtaining medication | Positive when financial, supply, or access difficulties were documented |
| Unintentional forgetfulness | 忘记用药；漏用；经常忘记 | Unintentional missed medication | Positive when missed medication was attributed to forgetting |
| Disease denial | 认为自己没有哮喘；不是哮喘；没有症状不需要用药 | Denial | Positive when the need for treatment or diagnosis was rejected |

**Table S3. Validation performance of the rule-enhanced clinical NLP framework and inter-physician agreement**

| **Barrier category** | **Cohen’s κ** | **Accuracy, %** | **PPV, %** | **Sensitivity, %** | **Specificity, %** | **F1 score** |
| --- | --- | --- | --- | --- | --- | --- |
| Intentional stop | 0.935 | 95.0 | 85.0 | 89.5 | 96.3 | 0.872 |
| Side effects | 0.795 | 100.0 | 100.0 | 100.0 | 100.0 | 1.000 |
| Access barrier | 0.870 | 93.0 | 72.0 | 100.0 | 91.5 | 0.837 |
| Unintentional Forgetfulness | 1.000 | 100.0 | 100.0 | 100.0 | 100.0 | 1.000 |
| Disease denial | 0.928 | 99.0 | 87.5 | 100.0 | 98.9 | 0.933 |
| Overall, pooled | 0.914 | 97.4 | 81.7 | 96.1 | 97.6 | 0.883 |

**Table S4. Characteristics and cumulative adherence barriers among patients with evaluable adherence data at Wave 3**

| **Variable** | **Total (n=176)** | **High adherence (n=114)** | **Low adherence (n=62)** | **P value** |
| --- | --- | --- | --- | --- |
| Age, years | 49.4 ± 16.5 | 48.3 ± 16.8 | 51.5 ± 15.9 | 0.265 |
| ACT score | 24.6 ± 0.8 | 24.7 ± 0.6 | 24.3 ± 1.0 | 0.031 |
| Sex |  |  |  | 0.520 |
| Female | 95 (54.0%) | 59 (51.8%) | 36 (58.1%) |  |
| Male | 81 (46.0%) | 55 (48.2%) | 26 (41.9%) |  |
| Cumulative adherence barriers |  |  |  |  |
| Intentional stop | 61 (34.7%) | 26 (22.8%) | 35 (56.5%) | <0.001 |
| Side effects fear | 34 (19.3%) | 10 (8.8%) | 24 (38.7%) | <0.001 |
| Access barrier | 61 (34.7%) | 44 (38.6%) | 17 (27.4%) | 0.186 |
| Unintentional forget | 10 (5.7%) | 2 (1.8%) | 8 (12.9%) | 0.007 |
| Disease denial | 34 (19.3%) | 16 (14.0%) | 18 (29.0%) | 0.027 |
